# Fast-nnt: fast, reproducible, and scalable neighbour network analysis in R, Python, and CLI

**DOI:** 10.1093/bioinformatics/btag577

**Published:** 2026-08-11

**Authors:** Rhys John Pembroke Newell, Eilish S McMaster

**Affiliations:** School of Life, Earth, and Environmental Sciences, University of Sydney, Camperdown, NSW, 2050, Australia; School of Life, Earth, and Environmental Sciences, University of Sydney, Camperdown, NSW, 2050, Australia

## Abstract

**Motivation:**

Neighbour networks are widely used to visualise evolutionary relationships in the presence of reticulation, admixture, or hybridization. Existing implementations are largely GUI-based, limiting reproducibility, integration into scripted workflows, and deployment on remote or high-performance computing systems. They are also computationally slow and memory-intensive at scale, restricting analyses to relatively small datasets. We present fast-nnt, an open-source Rust reimplementation of the neighbour-net algorithms from SplitsTree4 and SplitsTree6, providing interfaces for R, Python, and the command line.

**Results:**

fast-nnt is substantially faster and more memory-efficient than existing tools, completing analyses of 3333 taxa in ∼148 seconds compared to ∼1640 seconds for SplitsTree6, an 11-fold improvement, while using less than half the memory. It accepts any symmetric distance matrix and reproduces SplitsTree output with near-identical accuracy. Both the circular ordering algorithms (Multi-Way, Closest-Pair) and split weight inference methods (Conjugate Gradient, Active-Set) are independently selectable, enabling modular and reproducible analyses. This removes a major computational barrier to routine use of neighbour-net methods in large-scale phylogenetics. By addressing key computational and usability bottlenecks in existing implementations, fast-nnt enables scalable, reproducible neighbour-network inference that is practical for large modern datasets.

**Availability:**

Source code, documentation, and test data freely available at https://github.com/rhysnewell/fast-nnt. Implemented in Rust with R (fastnntr) and Python (fastnntpy) packages. An archived release is available at [https://doi.org/10.5281/zenodo.16907379]. Licensed under GNU General Public License v3.0.

## 1 Introduction

Neighbour networks are a powerful and flexible approach for visualizing evolutionary relationships when histories are non-tree-like, arising from hybridization, introgression, recombination, or incomplete lineage sorting (Mallet *et al*. 2016). Operating directly on any symmetric pairwise distance matrix, they produce implicit phylogenetic networks that simultaneously convey multiple, potentially conflicting signals, making them especially informative for identifying natural groupings while also representing uncertainty in a single visualization (Bryant and Moulton 2004, Morrison 2014). This makes them well suited to exploratory data analysis, in contrast to more computationally intensive explicit networks which model specific evolutionary processes directly (Kong *et al*. 2025).

Because neighbour networks are distance-based and model-free, they can be applied across biological scales, from genes and proteins (Lian *et al.* 2013, Tzlil *et al.* 2025), to whole genomes or individuals (Chen LY *et al.* 2019, McMaster *et al.* 2024), to higher-level groupings such as mitochondrial haplotypes (Paynee *et al.* 2026), and are less sensitive to distortion from clonal groups or family structure than ordination methods such as PCA or UMAP. These properties have made them valuable in population genomics (Smýkal *et al.* 2017, Kearns *et al.* 2018), conservation genomics (Kireta *et al.* 2019, Ambu *et al.* 2025, Klobučník *et al.* 2025, Paynee *et al.* 2026), microbiology (Chen LX *et al.* 2019, Heeren *et al.* 2023), and virology (Gao *et al.* 2019), as well as beyond molecular biology: neighbour networks have been applied to morphological data in palaeontology (Moon 2019), and to cultural datasets including manuscript traditions (Barbrook *et al.* 1998) and language dialects (Yang *et al.* 2024). Despite this breadth of application, adoption remains far from universal, and the method is underutilised relative to its analytical value.

The neighbour-net algorithm (Bandelt and Dress 1992, Bryant and Moulton 2004) is primarily accessed through the SplitsTree software suite (Huson and Bryant 2006, 2024), which provides mature and widely trusted implementations. However, SplitsTree is predominantly a graphical user interface (GUI) application. Typical usage involves exporting data to Nexus format, running the algorithm interactively, and re-importing results for downstream visualization in R or Python, a workflow that introduces unnecessary friction for researchers working in reproducible, script-based pipelines, and is incompatible with remote or high-performance computing environments. Although a command-line runner exists, it is sparsely documented and requires pre-configured workflow files generated through the GUI.

Scriptable alternatives exist but scale poorly. The *neighborNet* function in the *phangorn* R package (Schliep 2011) integrates directly into R workflows but becomes computationally intractable at the dataset sizes common in contemporary genomics. SplitsPy, a command-line Python tool from the SplitsTree developers, is similarly limited at moderate sample sizes. No existing tool combines fast, scalable performance with seamless integration into R or Python workflows.

**Figure 1:**
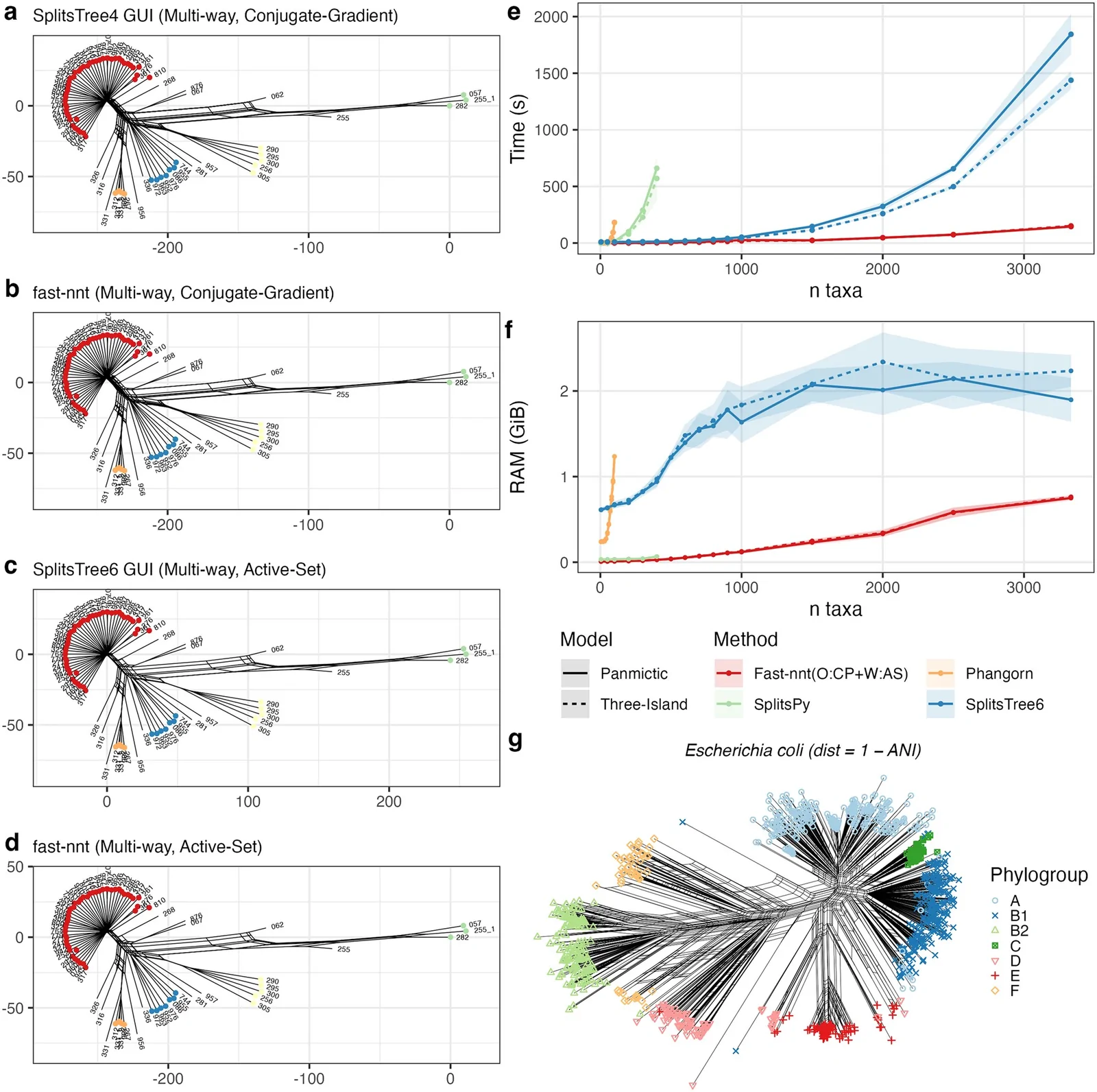
(a–d) Comparison of neighbour networks produced by the SplitsTree GUIs and fast-nnt reimplementations. The ordering and weight inference algorithms are indicated in brackets. The SplitsTree6 plot has *y* axis inverted from the default layout. (e, f) Runtime and memory performance of neighbour-net implementations. Elapsed time and peak RAM comparing fast-nnt against phangorn, SplitsPy, and SplitsTree6. The fastest fast-nnt configuration is shown (Closest-Pair ordering + Active-Set inference; all combinations in Supplementary 1). Lines show means ± SD across ten replicates (three for SplitsTree6 at *n* = 3333). (g) Network constructed from inverse ANI between 1377 *E. coli* GenBank assemblies. Using fast-nnt this layout took less than one minute directly in R. Reticulation among strains reflects the mosaic ancestry characteristic of bacterial evolution.

Here we present *fast-nnt*, a reimplementation of the SplitsTree4 and SplitsTree6 neighbour-net algorithms in Rust (Klabnik and Nichols 2023), with interfaces for R [via *rextendr* v0.8.1 (Wilke *et al.* 2025)] and Python via PyO3 v0.25.1 (PyO3 Project and Contributors 2023) and maturin v1.9.3 (Konstin 2026), as well as a standalone command-line interface. *fast-nnt* reproduces SplitsTree output with near-identical accuracy (Fig. 1a–d), is substantially faster and more memory-efficient at scale (Fig. 1e and f), and integrates directly into existing workflows without intermediate file formats or GUI interaction. To illustrate practical scalability, Fig. 1g shows a neighbour network constructed from inverse ANI distances among 1377 *E. coli* GenBank assemblies, produced directly in R in under one minute. By extending the usability of neighbour-networks to environments commonly used by analysts, fast-nnt makes neighbour-network analysis a routine and accessible method for any researcher working with pairwise dissimilarity data.

## 2 Algorithm and implementation

*fast-nnt* is a direct translation of the neighbour-net algorithms in the SplitsTree4 (https://github.com/husonlab/splitstree4) and SplitsTree6 (https://github.com/husonlab/splitstree6) public GitHub repositories (as of August 2025) from Java into Rust, preserving algorithmic structure and numerical behaviour. The neighbour-net algorithm proceeds in three stages:

- Circular ordering: Taxa are arranged into a circular order that approximately preserves pairwise distances, defining the planar embedding of the network.
- Split weight inference: Compatible and incompatible splits are identified and assigned weights from the input distances.
- Network layout: Weighted splits are converted to a two-dimensional layout with node coordinates and edge definitions suitable for visualization.

SplitsTree4 and SplitsTree6 differ primarily in the split weight inference step: SplitsTree4 uses Conjugate Gradient (CG) optimization, while SplitsTree6 implements the Active-Set (AS) method (Huson and Bryant 2006, Bryant and Huson 2023). SplitsTree6 also introduced a Closest-Pair (CP) circular ordering method alongside the existing Multi-Way (MW) approach, though the GUI exposes only MW by default. *fast-nnt* implements both ordering methods (MW and CP) and both inference methods (CG and AS) as independently selectable components. Users may therefore combine algorithms freely in *fast-nnt* (e.g. CP ordering with AS inference), improving transparency and enabling systematic comparison of algorithmic choices. Fast-nnt uses the equal-angle layout algorithm, as in SplitsTree (Kloepper and Huson 2008).

### 2.1 Performance optimizations

Beyond preserving algorithmic correctness, the *fast-nnt* Rust implementation introduces several targeted performance optimizations. The primary bottleneck in the split weight solver is repeated matrix-vector products within the CGNR iteration. The original Java code stores split weights in packed upper-triangular, row-major arrays; the diagonal-traversal recurrence causes variable-stride memory access and poor cache utilization. *fast-nnt* reorganises storage into a band-major layout, making all elements at the same diagonal offset contiguous and converting inner loops into stride-1 sweeps. This enables acceleration via platform-specific SIMD intrinsics (ARM NEON, x86 AVX, and SSE2) and fused kernel variants that accumulate the squared norm during the matrix-vector product, eliminating a redundant pass. The agglomerative ordering algorithms additionally use incremental maintenance of cluster-level statistics; distance sums and inter-cluster means are updated in place as clusters merge, rather than recomputed from the full distance matrix at every step as in the Java code.

### 2.2 Performance

We benchmarked *fast-nnt* against three existing implementations: *phangorn* (R), SplitsPy (CLI), and SplitsTree6 (CLI workflow runner). Haplotype data were simulated using *scrm* v1.7.5 (Staab *et al.* 2015) under a panmictic model and a three-island model with migration (μ  =  7 × 10^−9^, *r* = 3.5 × 10^−8^, sequence length = 20 Mbp, Ne = 3333, scaled migration rate = 0.8). For each method and dataset size, elapsed time and peak RAM were recorded across ten replicates (three for SplitsTree6 at *n* = 3333). Only the CLI version of *fast-nnt* was benchmarked, as the R and Python interfaces are FFI bindings to the same underlying Rust code.

At small scales (*n* ≤ 100), *fast-nnt* already outperforms all alternatives: completion in ∼0.02–0.28 s versus ∼0.9–21 s for SplitsPy, ∼8–13 s for SplitsTree6, and ∼0.8–187 s for *phangorn*. *phangorn* shows particularly poor scaling, runtime increases by more than two orders of magnitude between *n* = 30 and *n* = 100, and peak memory exceeds 1.3 GiB at *n* = 100, hence was not benchmarked beyond this size. SplitsPy reaches ∼615 s at *n* = 400. At larger scales, *fast-nnt* (using the fastest configuration: CP ordering with AS inference) completes *n* = 2500 in ∼74 s and *n* = 3333 in ∼148 s, representing an ∼8× and ∼11× speedup over SplitsTree6 respectively. Memory usage at *n* = 3333 is ∼766–959 MiB for *fast-nnt* versus ∼2–2.3 GiB for SplitsTree6 (Table 1).

**Table 1:** Comparison of neighbour network tools.

Tool	Interfaces	Input	Output	∼Time *n* = 100 (s)	∼Time *n* = 1000 (s)	∼Time *n* = 3333 (s)	Scriptable	File-free
phangorn	R	Dist. matrix	networx/phylo	∼181	—	—	✓	✓
SplitsPy	CLI	Dist. matrix	Splits (Python)	∼13	—	—	✓	×
SplitsTree6^a^	GUI	Nexus, dist. matrix	Nexus, stree6	∼9	∼48	∼1641	Partial	×
fast-nnt^b^	CLI, R, Python	CSV, TSV, Nexus	Nexus, networx/phylo	∼0.05–0.09	∼14–22	∼148–426	✓	✓

Approximate wall times are averages across replicates and demographic models.

a SplitsTree6 is primarily a GUI; CLI runner requires pre-configured workflow files from the GUI.

b Wall times show min–max across all ordering/weighting combinations.

Performance varies across algorithm combinations within *fast-nnt*: AS inference is consistently faster than CG regardless of ordering method, and CG shows greater runtime variance under MW ordering than under CP ordering. Full benchmarking results across all four algorithm combinations are provided in Supplementary 1.

## 3 Availability and implementation

fast-nnt is implemented in Rust (v1.89.0) with bindings for R (fastnntr; R ≥ 4.5.2) and Python (fastnntpy; Python ≥ 3.9), alongside a standalone CLI. The Python package installs from PyPI (pip install fastnntpy) and the R package from r-universe as a prebuilt binary (install.packages(“fastnntr”, repos = “https://rhysnewell.r-universe.dev”)), neither of which requires a Rust toolchain; the CLI installs from crates.io (cargo install fast-nnt). Building any component from source requires Rust via https://rustup.rs/. No additional system requirements are needed. The tool runs on macOS, Linux, and Windows, with continuous integration handling releases of Python packages across versions 3.9–3.14 on all three platforms. Continuous support is planned for future python versions as well.

Input is a symmetric, square distance matrix with labelled taxa or dist object; the distance metric is unrestricted. The primary functions are run_neighbornet_networkx() in R, run_neighbour_net() in Python, and neighbour_net in CLI. The ordering method and inference algorithm are independently selectable, and the default configuration (MW ordering + AS inference) reproduces SplitsTree6 output. Results are returned as in-memory objects in R (networx/phylo) and Python (node/edge lists), or written to SplitsTree-compatible Nexus files from the CLI. Parallelism is configurable via -t < threads> (CLI) or set_fastnnt_threads() (R/Python).

Source code, documentation, installation instructions, worked examples, and plotting guides are freely available at https://github.com/rhysnewell/fast-nnt (v0.3.0, released 16 June 2026) and archived at https://doi.org/10.5281/zenodo.16907379. Test coverage is 77.08% (3541/4594 lines), focused on the core algorithms rather than FFI bindings. fast-nnt is licensed under GPL-3.

## 4 Conclusion

*fast-nnt* makes neighbour network analysis fast, reproducible, and accessible at modern data scales. By reimplementing neighbour-net algorithms in Rust and exposing them through R, Python, and CLI interfaces, it eliminates the need for GUI interaction or intermediate file formats while delivering substantial improvements in speed and memory efficiency. Any researcher working with distance data can now integrate neighbour network analysis directly into scripted, reproducible pipelines. Issues, contributions, and feature requests are welcomed via the GitHub repository.

## Supplementary Material

btag577_Supplementary_Data

## Data Availability

Source code, documentation, and test data freely available at https://github.com/rhysnewell/fast-nnt.
