# Supplementary material for: Fast-nnt: fast, reproducible, and scalable neighbour network analysis in R, Python, and CLI
Source: Bioinformatics. 2026 Aug 11;42(8):btag577. doi: 10.1093/bioinformatics/btag577 (PMC13481693; doi:10.1093/bioinformatics/btag577)
Supplement: Supplementary file 1 [file btag577_supplementary_data.zip › fastnntr-vignette-RENDERED.pdf]

Introduction to fastnntr: Computing and Visualising Neighbour-Net Networks

Overview

`fastnntr` provides a fast interface to the Neighbour-Net algorithm directly in R. Given a pairwise distance matrix, it returns a network object that can be plotted with `phangorn` in base R or with `ggplot2`/`tanggle`.

This vignette walks through three self-contained examples:

1. **Genetic data** — SNP genotype matrix for a plant species (*Pherosphaera fitzgeraldii*)
2. **Genomic distances** — Average Nucleotide Identity (ANI) matrix for *Escherichia coli* isolates
3. **Morphological data** — discrete character matrix for pachycephalosaur dinosaurs

Why neighbour networks?

Neighbour networks were introduced almost three decades ago (Huson, 1998) but remain underutilised relative to their analytical value. Rather than replacing existing analyses, they synthesise information into an intuitive visual summary that can often be interpreted without specialist expertise. They have been applied in microbiology (Heeren et al., 2023; Lai & Ioerger, 2018), virology (Chen et al., 2010; Gao et al., 2019; Lian et al., 2013), and population genomics (Chen et al., 2019; Kearns et al., 2018; Smýkal et al., 2017).

A key strength of neighbour networks is their flexibility: because they operate directly on pairwise distance matrices, they can be applied across biological scales — from genes and proteins (Lian et al., 2013; Tzlil et al., 2025) to whole genomes (Chen et al., 2019; McMaster et al., 2024) to higher-level groupings such as mitochondrial haplotypes (Paynee et al., 2026). Their model-free representation of reticulation is especially valuable in systems with horizontal gene transfer or non-tree-like evolution (Mallet et al., 2016). In population genomics, a single network can simultaneously reveal population structure, clonal relationships, admixed individuals, and relative diversity, information that otherwise requires multiple complementary analyses (McMaster et al., 2024). Neighbour networks are also less sensitive to distortion from clonal groups or family structure than PCA or UMAP/t-SNE, making them a robust complementary visualisation tool.

They are equally applicable outside of genomics. In palaeontology and morphology-based disciplines, where model-based phylogenetic methods can be sensitive to missing or ambiguous data (Lamsdell et al., 2025; López-Antoñanzas et al., 2022), neighbour networks offer a model-free, immediately interpretable complement (Bomfleur et al., 2017; Gates & Scheetz, 2015). Beyond biology, they have been applied to manuscript traditions (Barbrook et al., 1998), folktales (Urban, 2025), musical instrument morphology (Aguirre-Fernández et al., 2021), and language dialects (Yang et al., 2024).

`fastnntr` enables neighbour network analysis in a fully reproducible, programmatic framework: analyses run directly from a distance matrix in R, results integrate into existing workflows, and visualisation is handled through `ggplot2` and `tanggle`. The three examples below illustrate the approach across genomic, whole-genome, and morphological data.

Installation

```r
# CRAN packages
cran_pkgs <- c("remotes", "ggplot2", "phangorn", "ape", "ggforce",
               "TreeSearch", "BiocManager")
install.packages(setdiff(cran_pkgs, rownames(installed.packages())))

# Bioconductor (tanggle and ggtree are distributed via Bioconductor)
bioc_pkgs <- c("ggtree", "tanggle")
bioc_need <- bioc_pkgs[!vapply(bioc_pkgs, requireNamespace, logical(1),
                               quietly = TRUE)]
if (length(bioc_need)) BiocManager::install(bioc_need)
```

```r
# fast-nnt itself
if (!requireNamespace("fastnntr", quietly = TRUE))
  remotes::install_git("https://github.com/rhysnewell/fast-nnt", subdir = "fastnntr")
```

```r
library(fastnntr)
library(phangorn)
library(tanggle)
library(ggplot2)
library(ggforce)
library(TreeSearch)
library(dplyr)
```

Example 1: Genetic data (SNP genotypes)

Input data

`PherFitz_gt` is a numeric matrix / data frame with **samples as rows** and **SNP loci as columns**. Each cell contains a dosage value (e.g. 0 / 1 / 2 for a diploid).

```r
PherFitz_gt   <- read.csv("data/PherFitz_gt.csv.gz",  row.names = 1)
PherFitz_meta <- read.csv("data/PherFitz_meta.csv.gz")

knitr::kable(PherFitz_gt[c(1,20,40,80),c(1,100,200)], format="markdown")
```

	X87883122.F.0.5.C.T.5.C.T	X87886069.F.0.8.A.G.8.A.G	X87891313.F.0.11.G.A.11.G.A
NSW1172053	NA	NA	2
NSW1171987	0	2	2
NSW1172112	2	0	1
NSW1171974	0	0	2

Distance matrix

We compute a standard Euclidean distance matrix and convert it to a plain numeric matrix. Any symmetric, non-negative distance matrix is accepted by `run_neighbornet_networkx()`.

```r
PherFitz_dist <- as.matrix(dist(PherFitz_gt, method = "euclidean"))

knitr::kable(PherFitz_dist[c(1,20,40,80),c(1,20,40,80)], format="markdown")
```

	NSW1172053	NSW1171987	NSW1172112	NSW1171974
NSW1172053	0.00000	12.27523	39.96580	40.99444
NSW1171987	12.27523	0.00000	40.92583	40.74353
NSW1172112	39.96580	40.92583	0.00000	29.82347
NSW1171974	40.99444	40.74353	29.82347	0.00000

Compute the Neighbour-Net

```r
PherFitz_nnet <- run_neighbornet_networkx(PherFitz_dist)
```

`run_neighbornet_networkx()` returns a list with:

Element	Description
`$translate`	Data frame mapping node indices to sample labels
`$.plot$vertices`	Matrix of x/y coordinates for every network vertex
`$.plot$edges`	Edge list (pairs of vertex indices)

Quick base-R plot

```
plot(PherFitz_nnet, cex=0.5, edge.width=0.5)
```

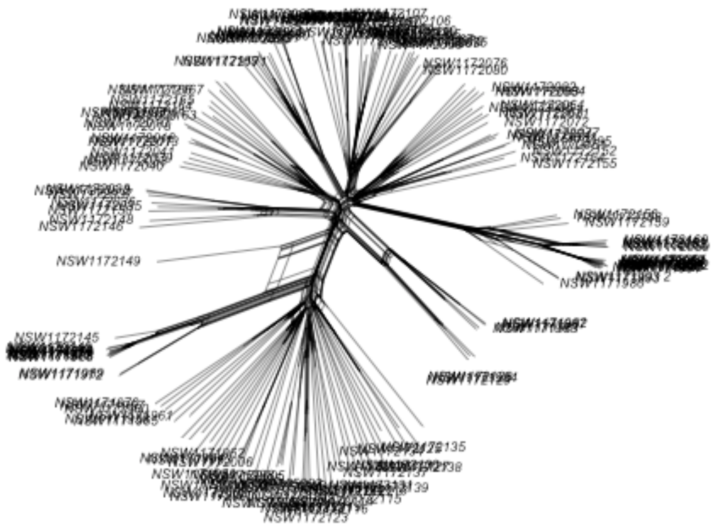

ggplot2 plot with tanggle

Attach metadata to the vertex coordinates so `ggplot2` can colour the tips by population. Clonal genets are circled in red.

```
# Build a data frame of tip coordinates with metadata
PherFitz_nnet_tips <- data.frame(
  x      = PherFitz_nnet$.plot$vertices[, 1],
  y      = PherFitz_nnet$.plot$vertices[, 2],
  sample = NA_character_
)
PherFitz_nnet_tips[PherFitz_nnet$translate$node, "sample"] <- PherFitz_nnet$translate$label
PherFitz_nnet_tips <- merge(PherFitz_nnet_tips, PherFitz_meta, by = "sample",
                All.x = TRUE, all.y = FALSE)

# Keep only rows that correspond to real samples (tips, not internal nodes)
PherFitz_nnet_tips2 <- PherFitz_nnet_tips[!is.na(PherFitz_nnet_tips$sample), ]

PherFitz_nnet_hull <- PherFitz_nnet_tips2 %>%
```

```
  filter(!is.na(genet)) %>%  # Remove rows with NA in genet, x, or y
  group_by(genet) %>%
  slice(chull(x, y))

ggplot(PherFitz_nnet, aes(x = x, y = y)) +
  geom_shape(data = PherFitz_nnet_hull,
             alpha = 0, expand = 0.01, radius = 0.01, color="red",
             aes(group=genet)) +
  geom_splitnet(layout = "slanted", linewidth = 0.2) +
  geom_point(data = PherFitz_nnet_tips2,
             aes(colour = pop_large_short, shape = pop_large_short),
             size = 1) +
  scale_shape_manual(values = 1:length(unique(PherFitz_nnet_tips2$pop_large_short)))+
  scale_colour_brewer(palette = "Paired", direction = -1) +
  coord_fixed() +
  theme_void() +
  labs(colour = "Population", shape = "Population", fill = "Population")
```

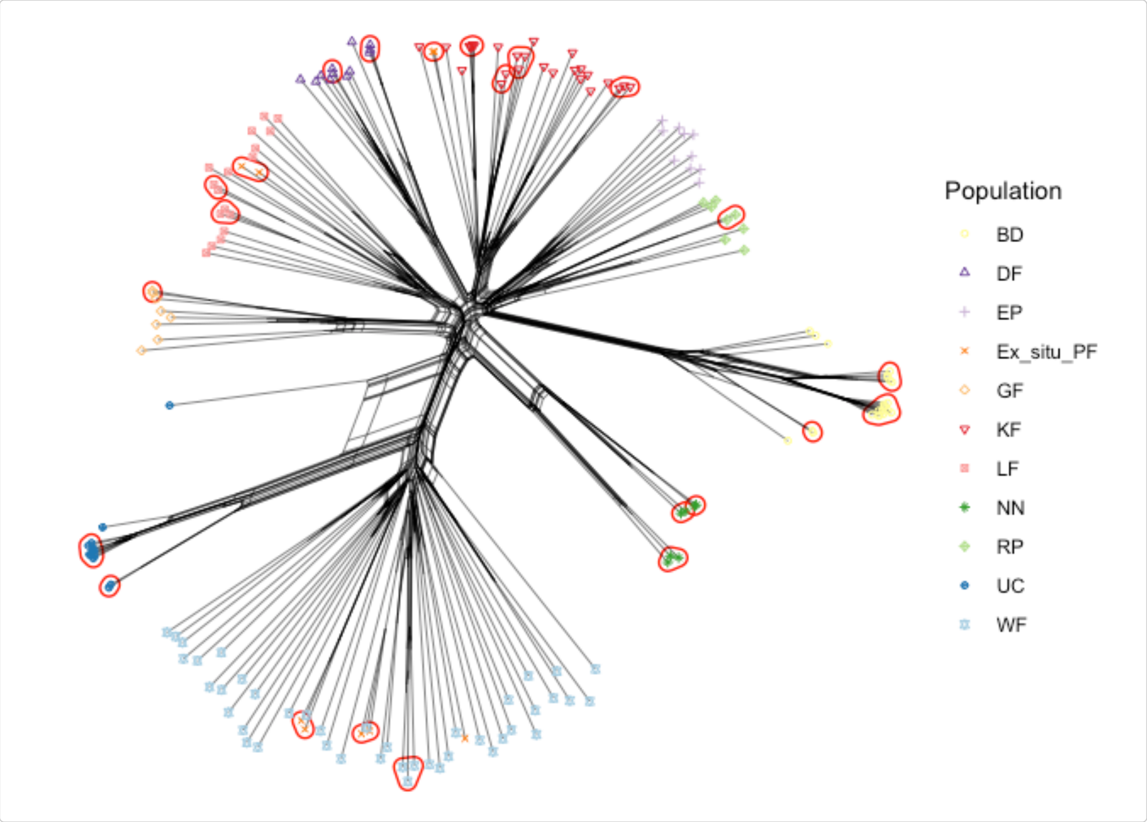

Network constructed among individuals of Pherosphaera fitzgeraldii derived from a biallelic SNP matrix (McMaster et al., 2024). Point colour and shape indicate populations. Clonal individuals are circled; these form visually distinct clusters with characteristically short branch lengths. Broader population-level structure is also clearly resolved.

Export

The network object is a plain R list and can be saved with `saveRDS()` or written to a Nexus-style splits file if your downstream tools require one.

```
saveRDS(PherFitz_nnet, file.path(tempdir(), "pherosphaera_nnet.rds"))

# write.nexus.networx(PherFitz_nnet, file = file.path(tempdir(), "pherosphaera_nnet.nexus"))
```

Example 2: Genomic distances (Average Nucleotide Identity)

ANI values are already pairwise distances; they need no further transformation before being passed to `run_neighbornet_networkx()`.

Input data

```
ani_names_raw <- read.csv("data/ecoli_dist_for_fastnnt.labels.txt.gz",
                          header = FALSE)
ani_names <- sub("_ASM.*", "", ani_names_raw[, 1])

ani_mx <- read.csv("data/ecoli_dist_for_fastnnt.tsv.gz",
                   sep = "\t", header = FALSE)
colnames(ani_mx) <- ani_names
rownames(ani_mx) <- ani_names

ani_meta <- read.csv("data/refseq_210120_mlst.tsv.gz", sep = "\t")
```

Filter to samples with known phylogroup

```
ani_meta <- subset(ani_meta, !Phylogroup %in% c("cladeI", "Unknown"))
keep     <- which(ani_names %in% ani_meta$genome)
ani_mx2  <- ani_mx[keep, keep]
```

Compute the Neighbour-Net

```
ani_nnet <- run_neighbornet_networkx(ani_mx2)
```

Optional: rotate the layout

The layout produced by Neighbour-Net is arbitrary up to reflection and rotation. You can apply any 2 × 2 rotation matrix to `$.plot$vertices` before plotting.

```
angle_rad <- -90 * pi / 180
R <- matrix(c(cos(angle_rad),  sin(angle_rad),
              -sin(angle_rad), cos(angle_rad)), ncol = 2)
ani_nnet$.plot$vertices <- as.matrix(ani_nnet$.plot$vertices) %*% R
```

ggplot2 plot

```
ani_nnet_tips <- data.frame(
  x      = ani_nnet$.plot$vertices[, 1],
  y      = ani_nnet$.plot$vertices[, 2],
  sample = NA_character_
)
ani_nnet_tips[ani_nnet$translate$node, "sample"] <- ani_nnet$translate$label

ani_meta$sample <- ani_meta$genome
ani_nnet_tips <- merge(ani_nnet_tips, ani_meta, by = "sample",
                       all.x = TRUE, all.y = FALSE)
ani_nnet_tips2 <- ani_nnet_tips[!is.na(ani_nnet_tips$sample), ]

ggplot(ani_nnet, aes(x = x, y = y)) +
  geom_splitnet(layout = "slanted", linewidth = 0.1) +
  geom_point(data = ani_nnet_tips2,
             aes(colour = Phylogroup, shape = Phylogroup),
             size = 1) +
  scale_colour_brewer(palette = "Paired", na.translate = FALSE) +
```

```r
  scale_shape_manual(
    values = seq_along(unique(ani_nnet_tips2$Phylogroup)),
    na.translate = FALSE
  ) +
  coord_fixed() +
  theme_void() +
  labs(colour = "Phylogroup", shape = "Phylogroup") +
  theme(legend.position = "right",
        legend.key.size  = unit(0.4, "lines"))
```

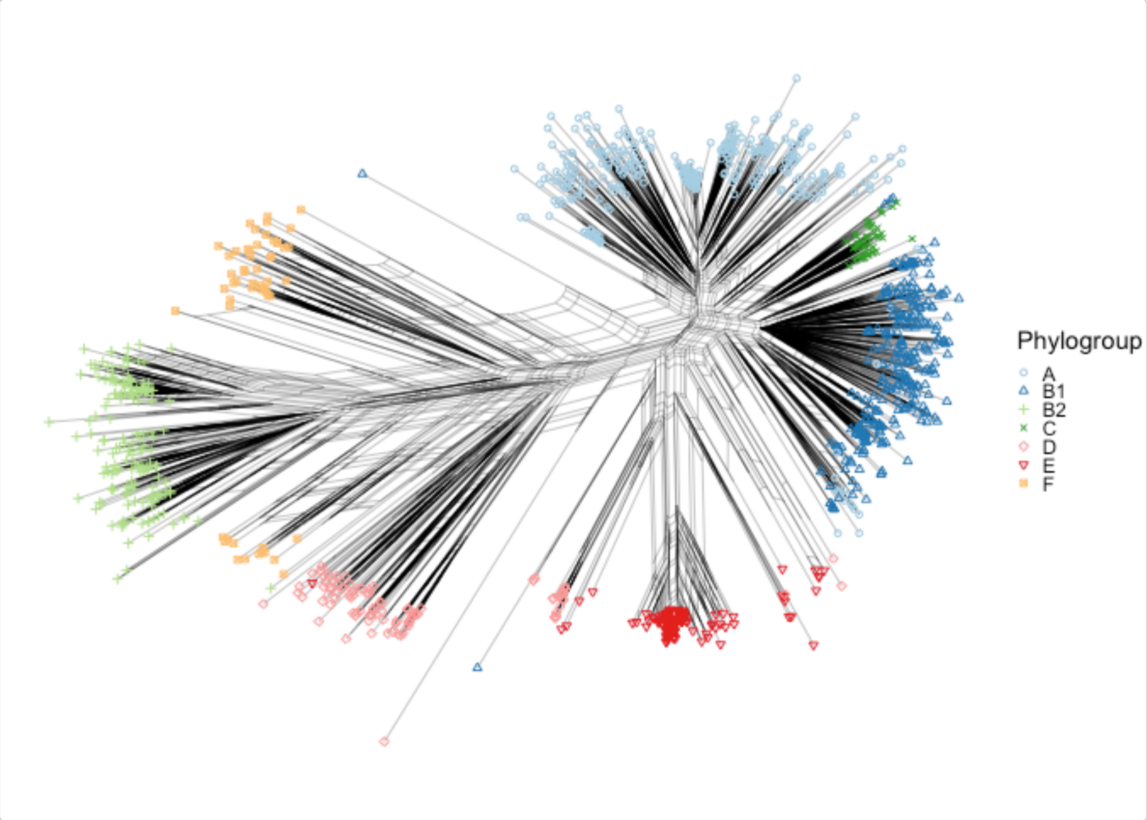

Network constructed from inverse ANI between 1,377 E. coli GenBank assemblies. Reticulation among strains reflects the mosaic ancestry characteristic of bacterial evolution.

Example 3: Morphological data (discrete characters)

Neighbour-Net is equally applicable to morphological character matrices. Here we use a published data set of pachycephalosaur dinosaurs bundled with the `TreeSearch` package.

Input data

```r
dino_morph <- as.data.frame(inapplicable.datasets$Longrich2010)

# Recode missing / inapplicable tokens to NA
dino_morph[dino_morph == "-"] <- NA
dino_morph[dino_morph == "?"] <- NA
```

Filter low-coverage taxa and characters

Remove characters missing in > 70 % of taxa, and taxa missing > 80 % of characters, to reduce noise in the distance matrix.

```r
dino_morph_filtered <- dino_morph[, colMeans(is.na(dino_morph)) < 0.7]
dino_morph_filtered <- dino_morph_filtered[rowMeans(is.na(dino_morph_filtered)) < 0.8, ]

knitr::kable(dino_morph_filtered[1:5,1:5], format="markdown")
```

Psittacosaurus_spp	Thescelosaurus_neglectus	Stegoceras_validum	Hanssuesia_sternbergi	Sphaerotholus_br
0	0	1	1	1
NA	NA	1	1	1
NA	NA	0	0	0
0	0	1	1	1
NA	NA	0	1	0

Distance matrix and network

We transpose so that **taxa are rows** before computing the distance.

```r
dino_morph_dist <- as.matrix(dist(t(dino_morph_filtered), method = "euclidean"))

dino_morph_nnet <- run_neighbornet_networkx(dino_morph_dist)

# Replace underscores with newlines for cleaner tip labels
dino_morph_nnet$translate$label <- gsub("_", "\n", dino_morph_nnet$translate$label)
```

ggplot2 plot

```r
ggplot(dino_morph_nnet) +
  geom_splitnet(linewidth = 0.2) +
  geom_tiplab2(size = 3, fontface = "italic", lineheight = 0.8) +
  scale_x_continuous(expand = c(0.3, 0.3)) +
  scale_y_continuous(expand = c(0.4, 0.4)) +
  coord_fixed() +
  theme_void()
```

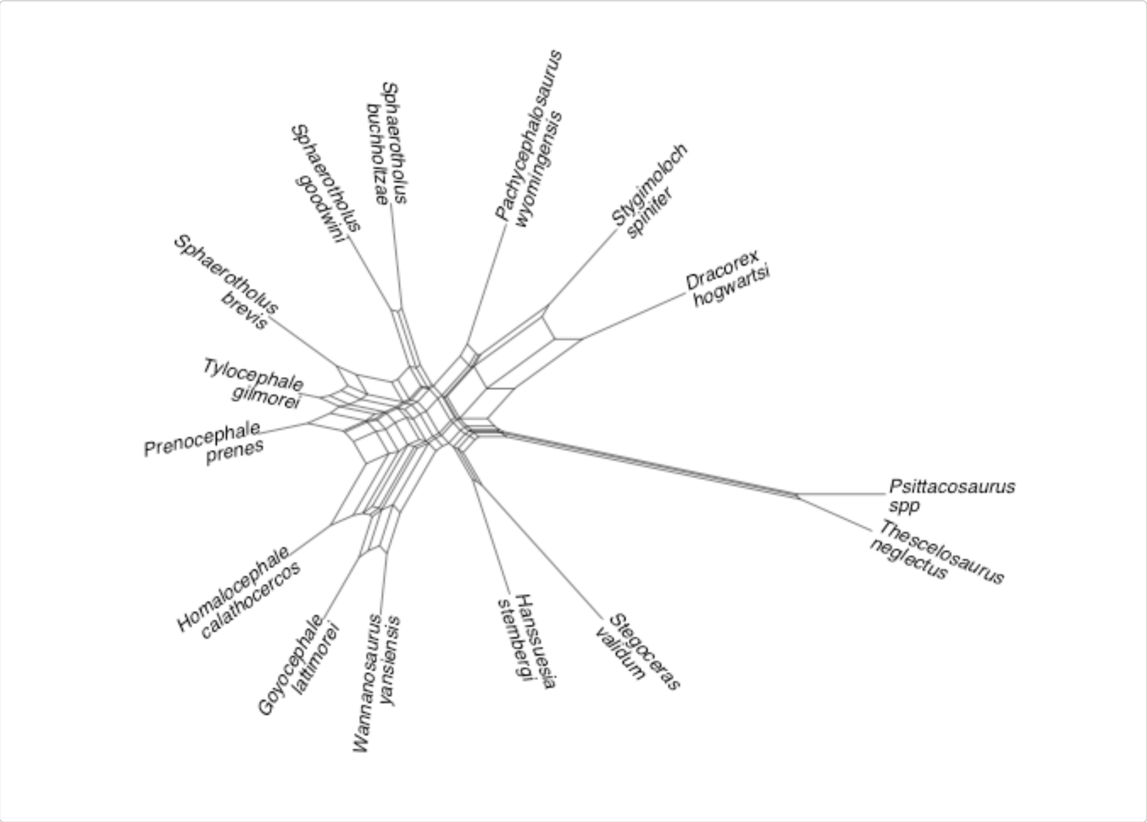

Network constructed morphological distances among Pachycephalosaur species (Longrich et al., 2010). Relationships are broadly congruent with the strict consensus tree reported in the original study, with the additional benefit that reticulation in the network reflects morphological ambiguity among taxa.

Summary of the core workflow

Every analysis follows the same three-step pattern:

```
distance matrix  →  run_neighbornet_networkx()  →  plot / export
```

The only required input is a square, symmetric, non-negative numeric matrix. The output is a standard R list whose key elements are documented in `?run_neighbornet_networkx`.

Session info

```
# sessionInfo()
```

References

Aguirre-Fernández, G., Barbieri, C., Graff, A., Pérez de Arce, J., Moreno, H., Sánchez-Villagra, M.R., 2021. Cultural macroevolution of musical instruments in South America. Humanit Soc Sci Commun 8, 208. https://doi.org/10.1057/s41599-021-00881-z

Ambu, J., Caballero-Díaz, C., Sánchez-Montes, G., Nicieza, A.G., Velo-Antón, G., Hernandez, A., Delmas, C., Trochet, A., Wielstra, B., Crochet, P.-A., Martínez-Solano, Íñigo, Dufresnes, C., 2025. Genome-wide patterns of diversity in the European midwife toad complex: phylogeographic and conservation prospects. Conserv Genet 26, 361–379. https://doi.org/10.1007/s10592-025-01673-7

Barbrook, A.C., Howe, C.J., Blake, N., Robinson, P., 1998. The phylogeny of The Canterbury Tales. Nature 394, 839–839. https://doi.org/10.1038/29667

Bomfleur, B., Grimm, G.W., McLoughlin, S., 2017. The fossil Osmundales (Royal Ferns)—a phylogenetic network analysis, revised taxonomy, and evolutionary classification of anatomically preserved trunks and rhizomes. PeerJ 5, e3433. https://doi.org/10.7717/peerj.3433

Chen, L.-Y., VanBuren, R., Paris, M., Zhou, H., Zhang, X., Wai, C.M., Yan, H., Chen, S., Alonge, M., Ramakrishnan, S., Liao, Z., Liu, J., Lin, J., Yue, J., Fatima, M., Lin, Z., Zhang, J., Huang, L., Wang, H., Hwa, T.-Y., Kao, S.-M., Choi, J.Y., Sharma, A., Song, J., Wang, L., Yim, W.C., Cushman, J.C., Paull, R.E., Matsumoto, T., Qin, Y., Wu, Q., Wang, J., Yu, Q., Wu, J., Zhang, S., Boches, P., Tung, C.-W., Wang, M.-L., Coppens d'Eeckenbrugge, G., Sanewski, G.M., Purugganan, M.D., Schatz, M.C., Bennetzen, J.L., Lexer, C., Ming, R., 2019. The bracteatus pineapple genome and domestication of clonally propagated crops. Nat Genet 51, 1549–1558. https://doi.org/10.1038/s41588-019-0506-8

Chen, X., Zhang, Q., Li, J., Cao, W., Zhang, J.-X., Zhang, L., Zhang, W., Shao, Z.-J., Yan, Y., 2010. Analysis of recombination and natural selection in human enterovirus 71. Virology 398, 251–261. https://doi.org/10.1016/j.virol.2009.12.007

Gao, F., Liu, X., Du, Z., Hou, H., Wang, X., Wang, F., Yang, J., 2019. Bayesian phylodynamic analysis reveals the dispersal patterns of tobacco mosaic virus in China. Virology 528, 110–117. https://doi.org/10.1016/j.virol.2018.12.001

Gates, T.A., Scheetz, R., 2015. A new saurolophine hadrosaurid (Dinosauria: Ornithopoda) from the Campanian of Utah, North America. Journal of Systematic Palaeontology 13, 711–725. https://doi.org/10.1080/14772019.2014.950614

Heeren, S., Maes, I., Sanders, M., Lye, L.-F., Adaui, V., Arevalo, J., Llanos-Cuentas, A., Garcia, L., Lemey, P., Beverley, S.M., Cotton, J.A., Dujardin, J.-C., Van den Broeck, F., 2023. Diversity and dissemination of viruses in pathogenic protozoa. Nat Commun 14, 8343. https://doi.org/10.1038/s41467-023-44085-2

Huson, D.H., 1998. SplitsTree: analyzing and visualizing evolutionary data. Bioinformatics 14, 68–73. https://doi.org/10.1093/bioinformatics/14.1.68

Jain, C., Rodriguez-R, L.M., Phillippy, A.M., Konstantinidis, K.T., Aluru, S., 2018. High throughput ANI analysis of 90K prokaryotic genomes reveals clear species boundaries. Nature Communications 9, 5114. https://doi.org/10.1038/s41467-018-07641-9

Kearns, A.M., Restani, M., Szabo, I., Schrøder-Nielsen, A., Kim, J.A., Richardson, H.M., Marzluff, J.M., Fleischer, R.C., Johnsen, A., Omland, K.E., 2018. Genomic evidence of speciation reversal in ravens. Nat Commun 9, 906. https://doi.org/10.1038/s41467-018-03294-w

Kireta, D., Christmas, M.J., Lowe, A.J., Breed, M.F., 2019. Disentangling the evolutionary history of three related shrub species using genome-wide molecular markers. Conserv Genet 20, 1101–1112. https://doi.org/10.1007/s10592-019-01197-x

Klobučník, M., van Oosterhout, C., Galgóci, M., Kormuťák, A., 2025. Exploring genetic admixture in putative hybrid zones of Pinus mugo Turra and P. sylvestris L. in Slovakia. Conserv Genet 26, 687–702. https://doi.org/10.1007/s10592-025-01696-0

Lai, Y.-P., Ioerger, T.R., 2018. A statistical method to identify recombination in bacterial genomes based on SNP incompatibility. BMC Bioinformatics 19, 450. https://doi.org/10.1186/s12859-018-2456-z

Lamsdell, J.C., Sheffield, S.L., Falk, A.R., 2025. A Practical Guide to Phylogenetic Paleoecology. Lian, S., Lee, J.-S., Cho, W.K., Yu, J., Kim, M.-K., Choi, H.-S., Kim, K.-H., 2013. Phylogenetic and Recombination Analysis of Tomato Spotted Wilt Virus. PLoS One 8, e63380. https://doi.org/10.1371/journal.pone.0063380

Longrich, N.R., Sankey, J., Tanke, D., 2010. Texacephale langstoni, a new genus of pachycephalosaurid (Dinosauria: Ornithischia) from the upper Campanian Aguja Formation, southern Texas, USA. Cretaceous Research 31, 274–284. https://doi.org/10.1016/j.cretres.2009.12.002

López-Antoñanzas, R., Mitchell, J., Simões, T.R., Condamine, F.L., Aguilée, R., Peláez-Campomanes, P., Renaud, S., Rolland, J., Donoghue, P.C.J., 2022. Integrative Phylogenetics: Tools for Palaeontologists to Explore the Tree of Life. Biology (Basel) 11, 1185. https://doi.org/10.3390/biology11081185

Mallet, J., Besansky, N., Hahn, M.W., 2016. How reticulated are species? BioEssays 38, 140–149. https://doi.org/10.1002/bies.201500149

McMaster, E.S., Yap, J.-Y.S., Chen, S.H., Sherieff, A., Bate, M., Brown, I., Jones, M., Rossetto, M., 2024. On the edge: Conservation genomics of the critically endangered dwarf mountain pine Pherosphaera fitzgeraldii. Basic and Applied Ecology 80, 61–71. https://doi.org/10.1016/j.baae.2024.09.003

Moon, B.C., 2019. A new phylogeny of ichthyosaurs (Reptilia: Diapsida). Journal of Systematic Palaeontology 17, 129–155. https://doi.org/10.1080/14772019.2017.1394922

Ondov, B.D., Treangen, T.J., Melsted, P., Mallonee, A.B., Bergman, N.H., Koren, S., Phillippy, A.M., 2016. Mash:

fast genome and metagenome distance estimation using MinHash. Genome Biol 17, 132. https://doi.org/10.1186/s13059-016-0997-x

Paynee, D., Vermeulen, E., Penry, G., Elwen, S., Matthee, C., Andreotti, S., Bloomer, P., 2026. Low genetic diversity and regional isolation of South Africa's inshore Bryde's whales. Conserv Genet 27, 26. https://doi.org/10.1007/s10592-025-01749-4

Serra Silva, A., 2024. Extended Lissamphibia: a tale of character non-independence, analytical parameters and islands of trees. Journal of Systematic Palaeontology 22, 2321620. https://doi.org/10.1080/14772019.2024.2321620

Shaw, J., Yu, Y.W., 2023. Fast and robust metagenomic sequence comparison through sparse chaining with skani. Nat Methods 20, 1661–1665. https://doi.org/10.1038/s41592-023-02018-3

Smýkal, P., Hradilová, I., Trněný, O., Brus, J., Rathore, A., Bariotakis, M., Das, R.R., Bhattacharyya, D., Richards, C., Coyne, C.J., Pirintsos, S., 2017. Genomic diversity and macroecology of the crop wild relatives of domesticated pea. Sci Rep 7, 17384. https://doi.org/10.1038/s41598-017-17623-4

Tzlil, G., Marín, M. del C., Matsuzaki, Y., Nag, P., Itakura, S., Mizuno, Y., Murakoshi, S., Tanaka, T., Larom, S., Konno, M., Abe-Yoshizumi, R., Molina-Márquez, A., Bárcenas-Pérez, D., Cheel, J., Koblížek, M., León, R., Katayama, K., Kandori, H., Schapiro, I., Shihoya, W., Nureki, O., Inoue, K., Rozenberg, A., Chazan, A., Béjà, O., 2025. Structural insights into light harvesting by antenna-containing rhodopsins in marine Asgard archaea. Nat Microbiol 10, 1484–1500. https://doi.org/10.1038/s41564-025-02016-5

Urban, M., 2025. How oral traditions develop: a cautionary tale on cultural evolution from the Quechuan-speaking Andes. Humanit Soc Sci Commun 12, 1604. https://doi.org/10.1057/s41599-025-05335-4

Yang, C., Zhang, X., Yan, S., Yang, S., Wu, B., You, F., Cui, Y., Xie, N., Wang, Z., Jin, L., Xu, S., Zhang, M., 2024. Large-scale lexical and genetic alignment supports a hybrid model of Han Chinese demic and cultural diffusions. Nat Hum Behav 8, 1163–1176. https://doi.org/10.1038/s41562-024-01886-9
